# An improved pairing-free certificateless aggregate signature scheme for healthcare wireless medical sensor networks

**DOI:** 10.1371/journal.pone.0268484

**Published:** 2022-07-11

**Authors:** Lifeng Zhou, Xinchun Yin

**Affiliations:** 1 College of Information Engineering, Yangzhou University, Yangzhou, Jiangsu, China; 2 College of Guangling, Yangzhou University, Yangzhou, Jiangsu, China; University College of Engineering Tindivanam, INDIA

## Abstract

In healthcare wireless medical sensor networks (HWMSNs), the medical sensor nodes are employed to collect medical data which is transmitted to doctors for diagnosis and treatment. In HWMSNs, medical data is vulnerable to various attacks through public channels. In addition, leakage of patients’ information happens frequently. Hence, secure communication and privacy preservation are major concerns in HWMSNs. To solve the above issues, Zhan et al. put forward a pairing-free certificateless aggregate signature (PF-CLAS) scheme. However, according to our cryptanalysis, the malicious medical sensor node (MSN_*i*_) can generate the forged signature by replacing the public key in the PF-CLAS scheme. Hence, to address this security flaw, we design the improved PF-CLAS scheme that can achieve unforgeability, anonymity, and traceability. Since we have changed the construction of the partial private key, the improved PF-CLAS scheme can resist Type I and Type II attacks under the Elliptic Curve Discrete Logarithm assumption. In terms of the performance evaluation, the proposed scheme outperforms related CLAS schemes, which is more suitable for HWMSNs environments.

## Introduction

With the rapid development of wireless body area networks, healthcare wireless medical sensor networks (HWMSNs) are driving the progress of intelligent medical treatment. In the current HWMSNs environment, patients use wearable and implantable medical devices from which multifarious medical data is collected [1]. Then the data is transmitted to doctors for real-time processing and feedback. Since the outbreak of COVID-19, hospitals have been using HWMSNs to monitor and treat the symptoms of patients [2]. However, medical data is transmitted through insecure public channels, and adversaries are able to eavesdrop on, tamper with, and forge the data readily [3, 4]. Upon tampering and forgery, doctors may make accurate diagnoses that can harm patients [5]. Furthermore, if the identities of patients are exposed in the form of plaintext, the patients’ real identities will be divulged [6–8]. Consequently, it is of great importance to guarantee secure communication and privacy preservation in HWMSNs.

In recent years, various technologies have been used for HWMSNs [9, 10]. To ensure the security of medical data, Mamta *et al*. [11] adopted the blockchain technology to design a decentralized and efficient attribute-based searchable encryption scheme. Nguyen *et al*. [12] put forward a blockchain-based intrusion detection and data transmission scheme that can realize the high-security level of the system. To guarantee secure communication and build trustworthiness among nodes in networks, Mirsadeghi *et al*. [13] presented a trust infrastructure-based authentication scheme by using digital signature and encryption technologies. Vijayakumar *et al*. [14] constructed a secure and lightweight communication scheme that can provide authentication and confidentiality to the multicast SMS communication. To achieve privacy preservation in HWMSNs, Xu *et al*. [15] proposed a sanitizable signature scheme that can hide the sensitive data of patients.

In 2003, a cryptographic technology called aggregate signature (AS) was proposed by Boneh *et al*. [16]. They showed that the AS can realize the authentication and integrity of the message with high efficiency, which makes it suitable for resource-constrained environments. Therefore, many authentication schemes using the AS have been proposed [17–22]. In 2004, Lysyanskaya *et al*. [17] constructed an ordered AS scheme based on a one-way function with trapdoors. Signers need to aggregate their signatures in the corresponding order. Whereas Lysyanskaya *et al*.’s scheme is based on the traditional public key infrastructure, which greatly increases the burden of key management and verification overhead. Soon after, Cheon *et al*. [18] proposed the first identity-based AS scheme that avoided complex certificate management issues. However, most identity-based AS schemes suffer from key escrow problems. Certificateless public key cryptography is considered one of the solutions to overcome these [23]. In [24], the full private key consists of the partial private key generated by the key generation center (KGC) and the secret value selected by the unmanned aerial vehicle. The aerial vehicle only knows its secret value and cannot achieve the partial private key of KGC. Hence, Gong *et al*. [20] extended the AS to certificateless public key cryptography and first proposed two certificateless AS (CLAS) schemes. Nevertheless, the complicated verification algorithm caused these two schemes to be inefficient.

Shortly after, the CLAS technology was widely applied to HWMSNs environments to address security and privacy problems. In 2018, Kumar *et al*. [25] designed a CLAS scheme to ensure the secure transmission of medical data in HWMSNs. Nevertheless, Wu *et al*. [26] proved that Kumar *et al*.’s scheme [25] is vulnerable to malicious medical server attacks. To ensure the high efficiency of verification and the identity privacy of patients, Liu *et al*. [27] devised a certificateless anonymous batch verification scheme and asserted that their scheme can authenticate all medical data in one time. Unfortunately, Zhang *et al*. [28] declared that Liu *et al*.’s scheme [27] is unable to withstand malicious participant attacks and malicious data center attacks. In 2019, Gayathri *et al*. [29] devised an anonymous CLAS scheme without bilinear pairings to further reduce the computational overhead. However, Liu *et al*. [30] substantiated that Gayathri *et al*.’s scheme [29] cannot withstand malicious MS attacks and public key replacement attacks. In addition, Liu *et al*. [30] proposed an improved scheme to resist the above attacks.

Recently, Zhan *et al*. [31] found that Liu *et al*.’s improved scheme [30] is insecure for the reason that it cannot withstand malicious MS attacks. To solve these security issues, Zhan *et al*. [31] put forward a pairing-free CLAS (PF-CLAS) scheme for HWMSNs. In addition, Zhan *et al*. [31] asserted that the PF-CLAS scheme has high computational efficiency and is secure against forgery attacks on any message. However, after our analysis, we found that the PF-CLAS scheme is unable to achieve the expected target.

### Contribution

The contributions of the proposed work are shown below.

1. We analyze Zhan *et al*.’s PF-CLAS scheme that cannot withstand malicious MSN_*i*_ attacks. Simultaneously, the process of how malicious MSN_*i*_ attacks successfully forge the signature is shown.
2. The reasons why Zhan *et al*.’s PF-CLAS scheme is insecure against malicious MSN_*i*_ attacks are explained. In addition, we design an improved PF-CLAS to address this security vulnerability.
3. We substantiate that our improved PF-CLAS scheme is secure under the random oracle model. Furthermore, the performance evaluation reveals that the proposed scheme is more efficient than the existing related schemes.

## Preliminaries

In this section, we introduce the complexity assumption, system model, security requirement, and security model in HWMSNs environments.

### Complexity assumption

#### Elliptic Curve Discrete Logarithm Problem (ECDLP)

The group *G* has the prime order *q* and generator *P*. Given two random points *P*, *Q* ∈ *G*, it is hard to work out $a\;\in \;Z_{q}^{*}$.

#### Computational Diffie-Hellman Problem (CDHP)

The group *G* has the order *q* and genera-tor *P*. Given two random points *aP*, *bP* ∈ *G*, it is hard to work out *abP* ∈ *G*, where $a,b\;\in \;Z_{q}^{*}$.

### System model of HWMSNs

**Fig 1:**
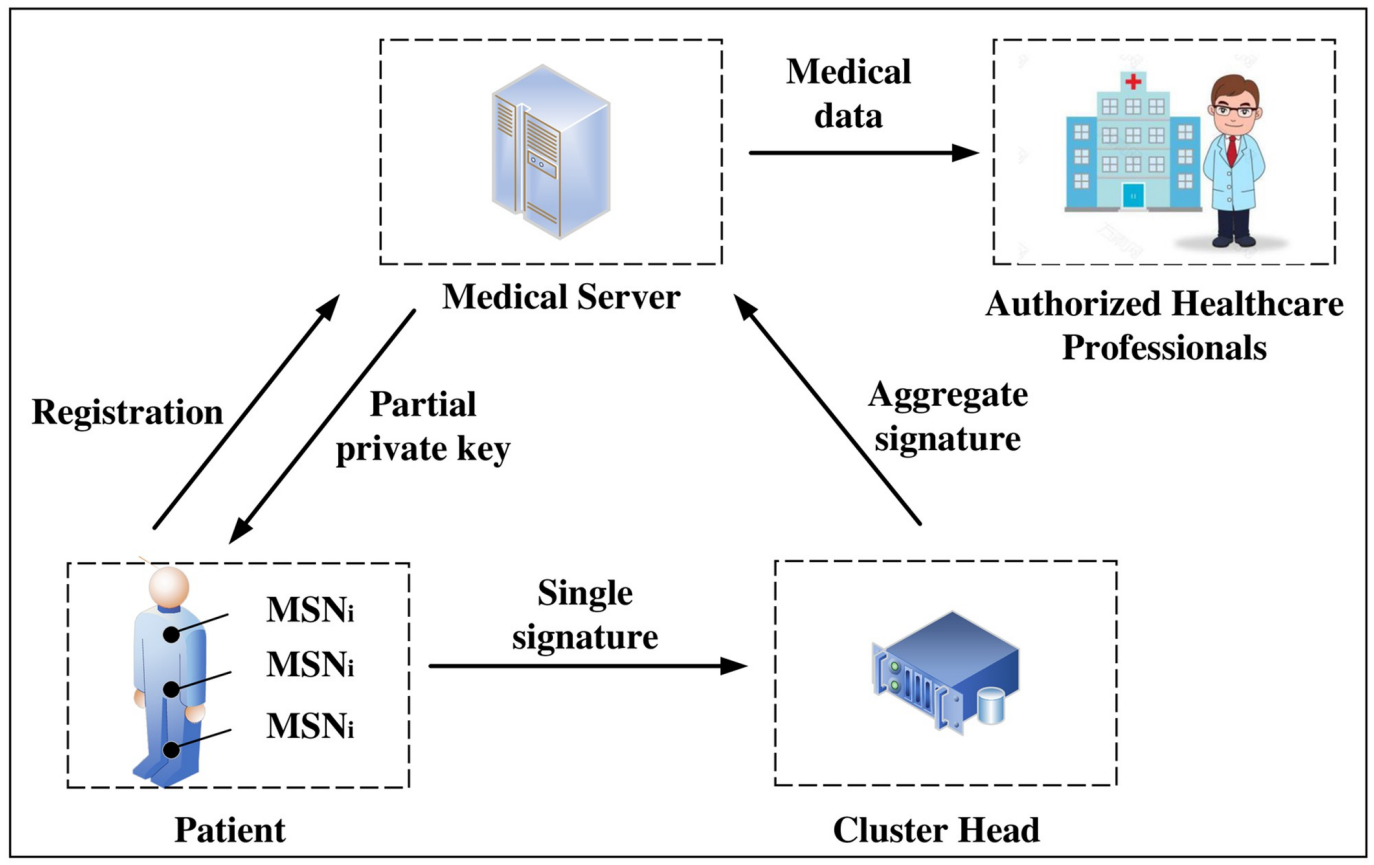
System model for HWMSNs.

As is described in Fig 1, the system model contains four entities: Medical Sensor Node (MSN_*i*_), Medical Server (MS), Cluster Head (CH), and Authorized Healthcare Professionals (AHP). MS is able to generate the public parameters and send it to MSN_*i*_. When MSN_*i*_ applies for the partial private key, MS will utilize the master secret key to generate the partial private key and send it to MSN_*i*_. Simultaneously, MSN_*i*_ takes advantage of its secret key and partial private key to create the signature and transmits it to CH. Multiple signatures can be aggregated into one signature by CH. Afterward, the aggregate signature can be transmitted to MS by CH. MS sends the aggregate signature to AHP after confirming the validity of the aggregate signature.

### Security requirements of HWMSNs

#### Message Authentication and Integrity

The messages received by the receiver are reliable and have not been tampered with during transmission.

#### Anonymity

No entity can know the real identity of MSN_*i*_ except MS and MSN_*i*_ itself.

#### Traceability

If abnormal MSN_*i*_ provides false medical data, MS will trace and extract the real identity of MSN_*i*_.

### Security model of HWMSNs

The CLAS scheme contains two types of adversaries: malicious MSN_*i*_ and malicious MS.

#### Malicious MSN_*i*_

It is Type I adversary $A_{1}$ in HWMSNs environments. Malicious MSN_*i*_ can replace the public key of MSN_*i*_, but it is incapable of achieving the master secret key *s*.

#### Malicious MS

It is Type II adversary $A_{2}$ in HWMSNs environments. Malicious MS can achieve the master secret key *s*, but it is incapable of replacing public keys.

The existential unforgeability of the PF-CLAS scheme is guaranteed by the following two games.

*Game 1*:

*Setup:* The *System Initialization* algorithm is executed by the challenger *ζ*_1_. Given the security parameter *v*, the algorithm returns system parameters *params* and master secret key *s*. *ζ*_1_ transmits *params* to $A_{1}$ while *s* is kept secretly.

*Query Phase:* $A_{1}$ carries out a bounded number of queries in polynomial time. The specific process is shown below.

- *PPK Query:* When $A_{1}$ makes queries on the partial private key with *PID*_*i*_, *ζ*_1_ returns *d*_*i*_ to $A_{1}$.
- *PK Query:* When $A_{1}$ makes queries on the public key of MSN_*i*_, *ζ*_1_ returns *PK*_*i*_ to $A_{1}$.
- *SV Query:* When $A_{1}$ makes queries on the secret value of MSN_*i*_ with *PID*_*i*_, *ζ*_1_ returns *x*_*i*_ to $A_{1}$.
- *PK Replacement Query:* When $A_{1}$ chooses a new public key $PK_{i}^{*}$ of MSN_*i*_ with *PID*_*i*_, *ζ*_1_ records this replacement.
- *Signature Query:* When $A_{1}$ makes queries on the signature with *PID*_*i*_ and *PK*_*i*_, *ζ*_1_ returns *σ*_*i*_ to $A_{1}$ in the tuple (*m*_*i*_, *PID*_*i*_, *PK*_*i*_).

*Forgery*: $A_{1}$ returns identities $\left\{PID_{1}^{*},\cdots ,PID_{n}^{*}\right\}$, public keys $\left\{PK_{1}^{*},\cdots ,PK_{n}^{*}\right\}$, messages $\left\{m_{1}^{*},\cdots ,m_{n}^{*}\right\}$, timestamps $\left\{t_{1}^{*},\cdots ,t_{n}^{*}\right\}$, and an AS σ*. $A_{1}$ can win *Game 1* if the following three situations happen:

1. σ* is a valid CLAS;
2. *PPK Query* has never been performed for at least one of $\left\{PID_{1}^{*},\cdots ,PID_{n}^{*}\right\}$;
3. *Signature Query* under the tuple $\left(PID_{i}^{*},m_{i}^{*},t_{i}^{*}\right)$ has never been performed, where 1 ≤ *i* ≤ *n*.

*Game 2*:

*Setup:* The *System Initialization* algorithm is executed by the challenger *ζ*_2_. Given the security parameter *v*, the algorithm returns system parameters *params* and master key *s*. *ζ*_2_ transmits *params* and *s* to $A_{2}$.

*Query Phase:* $A_{2}$ carries out a bounded number of queries in polynomial time. The specific process is shown below.

- *PK Query:* When $A_{2}$ makes queries on the public key of MSN_*i*_, *ζ*_2_ returns *PK*_*i*_ to $A_{2}$.
- *SV Query:* When $A_{2}$ makes queries on the secret value of MSN_*i*_ with *PID*_*i*_, *ζ*_2_ returns *x*_*i*_ to $A_{2}$.
- *Signature Query:* When $A_{2}$ makes queries on the signature with *PID*_*i*_ and *PK*_*i*_, *ζ*_2_ returns *σ*_*i*_ to $A_{2}$ in the tuple (*m*_*i*_, *PID*_*i*_, *PK*_*i*_).

*Forgery:* $A_{2}$ returns identities $\left\{PID_{1}^{*},\cdots ,PID_{n}^{*}\right\}$, public keys $\left\{PK_{1}^{*},\cdots ,PK_{n}^{*}\right\}$, messages $\left\{m_{1}^{*},\cdots ,m_{n}^{*}\right\}$, timestamps $\left\{t_{1}^{*},\cdots ,t_{n}^{*}\right\}$, and an AS σ*. $A_{2}$ can win *Game 2* if the following three situations happen:

1. σ* is a valid CLAS;
2. *SV Query* has never been performed for at least one of $\left\{PID_{1}^{*},\cdots ,PID_{n}^{*}\right\}$;
3. *Signature Query* under the tuple $\left(PID_{i}^{*},m_{i}^{*},t_{i}^{*}\right)$ has never been performed, where 1 ≤ *i* ≤ *n*.

## Review of PF-CLAS scheme in [31]

Here, we summarize the notations of the PF-CLAS scheme in Table 1 and review the PF-CLAS scheme in [31].

**Table 1:** Notations used in PF-CLAS scheme.

Notation	Description
*q*	A prime number
*P*	A generator of *G*
*s*	Master secret key
*P*_*pub*_	Master public key
*k*	Security parameter
*params*	System parameter
*RID*_*i*_	Real identity of MSN_*i*_
*PID*_*i*_	Pseudo identity of MSN_*i*_
*T*_*i*_	Valid time period of pseudo identity
*d*_*i*_	Partial private key of MSN_*i*_
*x*_*i*_	Secret value of MSN_*i*_
(*pk*_*i*_, *sk*_*i*_)	Public and private key pair of MSN_*i*_
*σ*	An aggregate signature
*t*_*i*_	Current timestamp

- **System Initialization** (1^*k*^) → (*params*): Given the security parameter $k\;\in \;Z_{q}^{*}$, MS performs the following procedures:
  - Selects an additive group *G* of order *q* and its generator *P*.
  - Selects $s\;\in \;Z_{q}^{*}$ as the master secret key at random and computes *P*_*pub*_ = *sP* as the master public key.
  - Selects hash functions: $H:G\times G\to Z_{q}^{*}$, $H_{1}:{\left\{0,1\right\}}^{*}\times G\times G\to Z_{q}^{*}$ and $H_{2}:{\left\{0,1\right\}}^{*}\times {\left\{0,1\right\}}^{*}\times G\times {\left\{0,1\right\}}^{*}\times G\to Z_{q}^{*}$.
  - Publishes *params* = {*P*, *G*, *q*, *P*_*pub*_, *H*, *H*_*i*, *i* = 1,2,3_} as the system parameter and keeps *s* secretly.
- **Generate-PPK** (*params*, *s*, *RID*_*i*_) → (*PID*_*i*_, *d*_*i*_): Given *s*, *RID*_*i*_ and *params*, MS performs the following procedures:
  - Selects $r_{i}\;\in \;Z_{q}^{*}$ randomly and calculates *R*_*i*_ = *r*_*i*_ *P*.
  - Computes *PID*_*i*_ = *RID*_*i*_⊕*H*(*r*_*i*_ *P*_*pub*_, *T*_*i*_), where *T*_*i*_ is the valid time period of *PID*_*i*_.
  - Computes *l*_*i*_ = *H*_1_(*R*_*i*_, *PID*_*i*_, *P*_*pub*_), *d*_*i*_ = (*r*_*i*_ + *sl*_*i*_) mod *q*.
  - Sets *D*_*i*_ = (*d*_*i*_, *R*_*i*_) as the private key and sends (*D*_*i*_, *PID*_*i*_) to MSN_*i*_ through secure channels.
- **Generate-PK/SK** (*params*, *PID*_*i*_, *d*_*i*_) → (*pk*_*i*_, *sk*_*i*_): Given *params*, *PID*_*i*_ and *d*_*i*_, MSN_*i*_ performs the following procedures:
  - Verifies whether the equation *d*_*i*_ *P* = *R*_*i*_ + *l*_*i*_ *P*_*pub*_ holds, if it holds, MSN_*i*_ accepts the private key *d*_*i*_. Otherwise, it needs to reapply to MS for the partial private key.
  - Selects $x_{i}\;\in \;Z_{q}^{*}$ randomly and calculates *X*_*i*_ = *x*_*i*_ *P*.
  - Sets *pk*_*i*_ = (*R*_*i*_, *X*_*i*_) as its own public key and *sk*_*i*_ = (*d*_*i*_, *x*_*i*_) as its own private key.
- **Generate-Signature** (*params*, *PID*_*i*_, *pk*_*i*_, *sk*_*i*_, *m*_*i*_, *t*_*i*_) → (*σ*_*i*_): Given *params*, *PID*_*i*_, *pk*_*i*_, *sk*_*i*_, a message *m*_*i*_ and timestamp *t*_*i*_, MSN_*i*_ performs the following procedures:
  - Chooses $y_{i}\;\in \;Z_{q}^{*}$ randomly and calculates *Y*_*i*_ = *y*_*i*_ *P*.
  - Calculates $a_{i}=H_{2}\left(PID_{i},m_{i},t_{i},Y_{i},pk_{i}\right)$ and *b*_*i*_ = *H*_3_(*PID*_*i*_, *m*_*i*_, *t*_*i*_, *pk*_*i*_).
  - Calculates *w*_*i*_ = [*a*_*i*_ *y*_*i*_ + *b*_*i*_(*d*_*i*_ + *x*_*i*_)] mod *q*.
  - Outputs *σ*_*i*_ = (*Y*_*i*_, *w*_*i*_) and transmits (*σ*_*i*_, *m*_*i*_, *t*_*i*_, *pk*_*i*_) to CH through public channels.
- **Verify-Signature** (*params*, *pk*_*i*_, {*m*_*i*_, *t*_*i*_})→ **VALID** or **INVALID**: Given *params*, *pk*_*i*_ and a set of message signature pairs (*m*_*i*_, *σ*_*i*_), CH performs the following procedures:
  - Computes *l*_*i*_ = *H*_1_(*R*_*i*_, *PID*_*i*_, *P*_*pub*_), *a*_*i*_ = *H*_2_(*PID*_*i*_, *m*_*i*_, *t*_*i*_, *Y*_*i*_, *pk*_*i*_) and *b*_*i*_ = *H*_3_(*PID*_*i*_, *m*_*i*_, *t*_*i*_, *pk*_*i*_).
  - Verifies whether the equation *W*_*i*_−*a*_*i*_ *Y*_*i*_ = *b*_*i*_(*X*_*i*_ + *R*_*i*_ + *l*_*i*_ *P*_*pub*_) holds, if it holds, CH outputs **VALID** and accepts the signature. Otherwise, CH outputs **INVALID** and rejects the signature.
- **Generate-AS** (*params*, *pk*_*i*_, {*m*_*i*_, *t*_*i*_, *σ*_*i*_}_1 ≤ *i* ≤ *n*_) → (*σ*): Given *params* and a set of message signature pairs (*m*_*i*_, *σ*_*i*_), CH performs the following procedures:
  - Computes $a_{i}=H_{2}\left(PID_{i},m_{i},t_{i},Y_{i},pk_{i}\right)$.
  - Computes $A=\sum_{i=1}^{n}a_{i}Y_{i}$.
  - Computes $w=\sum_{i=1}^{n}w_{i}$.
  - Outputs an aggregate signature *σ* = (*A*, *w*) and transmits (*σ*, *m*_*i*_, *t*_*i*_, *pk*_*i*_) to MS through public channels.
- **Verify-AS** (*params*, {*m*_*i*_, *t*_*i*_}_1 ≤ *i* ≤ *n*_, *σ*) → **VALID** or **INVALID**: Given *params*, *pk*_*i*_, {*m*_*i*_, *t*_*i*_}_1 ≤ *i* ≤ *n*_ and *σ*, MS performs the following procedures:
  - Computes *l*_*i*_ = *H*_1_(*R*_*i*_, *PID*_*i*_, *P*_*pub*_), *a*_*i*_ = *H*_2_(*PID*_*i*_, *m*_*i*_, *t*_*i*_, *Y*_*i*_, *pk*_*i*_) and *b*_*i*_ = *H*_3_(*PID*_*i*_, *m*_*i*_, *t*_*i*_, *pk*_*i*_), where 1 ≤ *i* ≤ *n*.
  - Checks whether the equation $wP-A=\sum_{i=1}^{n}\left[b_{i}\left(X_{i}+R_{i}+l_{i}P_{pub}\right)\right]$ holds. If it holds, MS outputs **VALID** and accepts the aggregate signature *σ*. Otherwise, MS outputs **INVALID** and rejects the aggregate signature *σ*.

## Cryptanalysis of PF-CLAS schemes

In this section, we first describe the detailed process of malicious MSN_*i*_ attacks, and then show the reason why this scheme cannot resist this type of attack. Finally, we present methods to withstand malicious MSN_*i*_ attacks.

### Forgery attacks from malicious MSN_*i*_

Although malicious MSN_*i*_ hardly gets the master key *s*, it can replace the public key *pk*_*i*_. In addition, if malicious MSN_*i*_ eliminates *l*_*i*_ *P*_*pub*_ by replacing the public key *pk*_*i*_, then it will bypass the system master key *s* to forge a valid signature. Malicious MSN_*i*_ can forge the valid signature on any stochastically chosen message $m_{i}^{*}$ that satisfies the condition $m_{i}^{*}\neq m_{i}$. The concrete descriptions are shown below.

1. **Public Key Replacement:** Malicious MSN_*i*_ executes the following procedures to replace the original public key *pk*_*i*_.
  - Selects $x_{i}^{{}^{\prime }}\;\in \;Z_{q}^{*}$ and $r_{i}^{{}^{\prime }}\;\in \;Z_{q}^{*}$ randomly.
  - Calculates $R_{i}^{{}^{\prime }}=r_{i}^{{}^{\prime }}P$ and $l_{i}=H_{1}\left(PID_{i},R_{i}^{{}^{\prime }},P_{pub}\right)$, where *PID*_*i*_ and *P*_*pub*_ are public.
  - Computes $X_{i}^{{}^{\prime }}=x_{i}^{{}^{\prime }}P-l_{i}P_{pub}$ to replace *X*_*i*_ and sets $pk_{i}^{{}^{\prime }}=\left(R_{i}^{{}^{\prime }},X_{i}^{{}^{\prime }}\right)$ as the new public key.
2. **Forgery:** Malicious MSN_*i*_ executes the following procedures to forge the signature $\sigma_{i}^{{}^{\prime }}$.
  - Chooses $y_{i}^{{}^{\prime }}\;\in \;Z_{q}^{*}$ and computes $Y_{i}^{{}^{\prime }}=y_{i}^{{}^{\prime }}P$.
  - Computes $a_{i}=H_{2}\left(PID_{i},m_{i}^{*},t_{i}^{*},Y_{i}^{{}^{\prime }},pk_{i}^{{}^{\prime }}\right)$, $b_{i}=H_{3}\left(PID_{i},m_{i}^{*},t_{i}^{*},pk_{i}^{{}^{\prime }}\right)$ and $w_{i}^{{}^{\prime }}=\left[a_{i}y_{i}^{{}^{\prime }}+b_{i}\left(x_{i}^{{}^{\prime }}+r_{i}^{{}^{\prime }}\right)\right]\;\text{mod}\;q$.
  - Sets $\sigma_{i}^{{}^{\prime }}=\left(Y_{i}^{{}^{\prime }},w_{i}^{{}^{\prime }}\right)$ as the forged signature and sends $<PID_{i},pk_{i}^{{}^{\prime }},m_{i}^{*}\|t_{i}^{*},\sigma_{i}^{{}^{\prime }}>$ to CH.
3. **Verification:** CH executes the following procedures to check the validity of the forged signature $\sigma_{i}^{{}^{\prime }}$.
  - Calculates $a_{i}=H_{2}\left(PID_{i},m_{i}^{*},t_{i}^{*},Y_{i}^{{}^{\prime }},pk_{i}^{{}^{\prime }}\right)$, $l_{i}=H_{1}\left(PID_{i},P_{pub},R_{i}^{{}^{\prime }}\right)$ and $b_{i}=H_{3}\left(PID_{i},m_{i}^{*},t_{i}^{*},pk_{i}^{{}^{\prime }}\right)$.
  - Checks whether the equation $w_{i}^{{}^{\prime }}P-a_{i}Y_{i}^{{}^{\prime }}=b_{i}\left(X_{i}^{{}^{\prime }}+R_{i}^{{}^{\prime }}+l_{i}P_{pub}\right)$ holds. If the equation holds, CH takes over the forged signature. Otherwise, malicious MSN_*i*_ fails to forge the signature.
4. **Correctness of the Forged Signature:** The validity of forged signature $\sigma_{i}^{{}^{\prime }}$ is supported by the verifiable equation.
  $$\begin{array}{cc} w_{i}^{{}^{\prime }}P-a_{i}Y_{i}^{{}^{\prime }} & =\left[a_{i}y_{i}^{{}^{\prime }}+b_{i}\left(x_{i}^{{}^{\prime }}+r_{i}^{{}^{\prime }}\right)\right]P-a_{i}Y_{i}^{{}^{\prime }}\\ & =a_{i}y_{i}^{{}^{\prime }}P+b_{i}\left(x_{i}^{{}^{\prime }}P+r_{i}^{{}^{\prime }}P\right)-a_{i}Y_{i}^{{}^{\prime }}\\ & =a_{i}y_{i}^{{}^{\prime }}P+b_{i}\left(x_{i}^{{}^{\prime }}P+R_{i}^{{}^{\prime }}\right)-a_{i}Y_{i}^{{}^{\prime }}\\ & =a_{i}Y_{i}^{{}^{\prime }}+b_{i}\left(x_{i}^{{}^{\prime }}P+R_{i}^{{}^{\prime }}\right)-a_{i}Y_{i}^{{}^{\prime }}\\ & =a_{i}Y_{i}^{{}^{\prime }}+b_{i}\left(X_{i}^{{}^{\prime }}+R_{i}^{{}^{\prime }}+l_{i}P_{pub}\right)-a_{i}Y_{i}^{{}^{\prime }}\\ & =b_{i}\left(X_{i}^{{}^{\prime }}+R_{i}^{{}^{\prime }}+l_{i}P_{pub}\right). \end{array}$$

### Comments on the reason for malicious MSN_*i*_ attacks

Although Zhan *et al*.’s scheme [31] has strived to solve the vulnerabilities of Liu *et al*.’s scheme in [30], it still suffers from malicious MSN_*i*_ attacks. In Zhan *et al*.’s PF-CLAS scheme [31], there’s no connection between *d*_*i*_ and *X*_*i*_, which is the main reason why malicious MSN_*i*_ can succeed in launching public key replacement attacks. The partial private key is defined as *d*_*i*_ = *r*_*i*_+ *sl*_*i*_ in the literature [31], where *l*_*i*_ = *H*_1_(*R*_*i*_, *PID*_*i*_, *P*_*pub*_). We can easily find that hash function *l*_*i*_ does not contain the public key *X*_*i*_, implying that the change of *X*_*i*_ cannot influence the partial private key *d*_*i*_. Hence, malicious MSN_*i*_ can bypass *d*_*i*_ by replacing *X*_*i*_ with $X_{i}^{{}^{\prime }}=x_{i}^{{}^{\prime }}P-l_{i}P_{pub}$. To avoid the public key replacement attacks launched by malicious MSN_*i*_, we only need to add the element *X*_*i*_ to hash functions *l*_*i*_ in **Generate-PPK** algorithm. After modification, it is obvious that the equation *d*_*i*_ *P* = *R*_*i*_ + *l*_*i*_ *P*_*pub*_ will not be valid if the public key *X*_*i*_ is replaced by adversaries, where *l*_*i*_ = *H*_1_(*R*_*i*_, *PID*_*i*_, *P*_*pub*_, *X*_*i*_).

## Improved PF-CLAS scheme

In this section, we devise an improved PF-CLAS scheme to avoid malicious MSN_*i*_ attacks in HWMSNs. The detailed algorithms are shown as follows.

- **System Initialization** (1^*k*^)→(*params*): Given the security parameter $k\;\in \;Z_{q}^{*}$, MS performs the following procedures:
  - Selects an additive group *G* of order *q* and its generator *P*.
  - Selects $s\;\in \;Z_{q}^{*}$ as the master secret key at random and computes *P*_*pub*_ = *sP* as the master public key.
  - Selects hash functions: $H:G\times {\left\{0,1\right\}}^{*}\to Z_{q}^{*}$, $H_{1}:G\times {\left\{0,1\right\}}^{*}\times G\times G\to Z_{q}^{*}$, $H_{2}:{\left\{0,1\right\}}^{*}\times {\left\{0,1\right\}}^{*}\times {\left\{0,1\right\}}^{*}\times G\times G\to Z_{q}^{*}$ and $H_{3}:{\left\{0,1\right\}}^{*}\to Z_{q}^{*}$.
  - Publishes *params* = {*P*, *G*, *q*, *P*_*pub*_, *H*, *H*_*i*, *i* = 1,2,3_} as the system parameter and keeps *s* secretly.
- **Generate-SV** (*params*)→(*x*_*i*_, *X*_*i*_): Given *params*, MSN_*i*_ performs the following procedures:
  - Selects $x_{i}\;\in \;Z_{q}^{*}$ randomly and calculates *X*_*i*_ = *x*_*i*_ *P*.
  - Transmits *X*_*i*_ to MS through public channels.
- **Generate-PPK** (*params*, *s*, *RID*_*i*_, *X*_*i*_)→(*PID*_*i*_, *d*_*i*_): Given *s*, *RID*_*i*_ and *params*, MS performs the following procedures:
  - Selects $r_{i}\;\in \;Z_{q}^{*}$ randomly and calculates *R*_*i*_ = *r*_*i*_ *P*.
  - Computes *PID*_*i*_ = *RID*_*i*_⊕*H*(*r*_*i*_ *P*_*pub*_, *T*_*i*_), *l*_*i*_ = *H*_1_(*R*_*i*_, *PID*_*i*_, *P*_*pub*_, *X*_*i*_) and *d*_*i*_ = (*r*_*i*_ + *sl*_*i*_) mod *q*.
  - Sets *D*_*i*_ = (*d*_*i*_, *R*_*i*_) as the private key and sends (*D*_*i*_, *PID*_*i*_) to MSN_*i*_ through secure channels.
- **Generate-PK/SK** (*params*, *PID*_*i*_, *d*_*i*_)→(*pk*_*i*_, *sk*_*i*_): Given *params*, *PID*_*i*_ and *d*_*i*_, MSN_*i*_ performs the following procedures:
  - Verifies whether the equation *d*_*i*_ *P* = *R*_*i*_ + *l*_*i*_ *P*_*pub*_ holds, if it holds, MSN_*i*_ accepts the private key *d*_*i*_. Otherwise, it needs to reapply to MS for the partial private key.
  - Sets *pk*_*i*_ = (*R*_*i*_, *X*_*i*_) as its own public key and *sk*_*i*_ = (*d*_*i*_, *x*_*i*_) as its own private key.
- **Generate-Signature** (*params*, *PID*_*i*_, *pk*_*i*_, *sk*_*i*_, *m*_*i*_, *t*_*i*_)→(*σ*_*i*_): Given *params*, *PID*_*i*_, *pk*_*i*_, *sk*_*i*_, a message *m*_*i*_ and timestamp *t*_*i*_, MSN_*i*_ performs the following procedures:
  - Chooses $y_{i}\;\in \;Z_{q}^{*}$ randomly and calculates *Y*_*i*_ = *y*_*i*_ *P*.
  - Calculates *b*_*i*_ = *H*_2_(*PID*_*i*_, *m*_*i*_, *t*_*i*_, *pk*_*i*_, *Y*_*i*_) and *w*_*i*_ = [*y*_*i*_ + *b*_*i*_(*d*_*i*_ + *x*_*i*_)] mod *q*.
  - Outputs *σ*_*i*_ = (*Y*_*i*_, *w*_*i*_) and transmits (*σ*_*i*_, *m*_*i*_, *t*_*i*_, *pk*_*i*_) to CH through public channels.
- **Verify-Signature** (*params*, *pk*_*i*_, {*m*_*i*_, *t*_*i*_}) → **VALID** or **INVALID**: Given *params*, *pk*_*i*_ and a set of message signature pairs (*m*_*i*_, *σ*_*i*_), CH performs the following procedures:
  - Computes *l*_*i*_ = *H*_1_(*R*_*i*_, *PID*_*i*_, *P*_*pub*_, *X*_*i*_), *b*_*i*_ = *H*_2_(*PID*_*i*_, *m*_*i*_, *t*_*i*_, *pk*_*i*_, *Y*_*i*_).
  - Verifies whether the equation *W*_*i*_−*Y*_*i*_ = *b*_*i*_(*X*_*i*_ + *R*_*i*_ + *l*_*i*_ *P*_*pub*_) holds, if it holds, CH outputs **VALID** and accepts the signature. Otherwise, CH outputs **INVALID** and rejects the signature.
- **Generate-AS** (*params*, *pk*_*i*_, {*m*_*i*_, *t*_*i*_, *σ*_*i*_}_1 ≤ *i* ≤ *n*_, *pk*_*ver*_)→(*σ*): Given *params*, *pk*_*ver*_ and the tuple (*σ*_*i*_, *m*_*i*_, *t*_*i*_), CH performs the following procedures:
  - Computes $w=\sum_{i=1}^{n}w_{i}$.
  - Outputs an aggregate signature *σ* = (*Y*_1_, *Y*_2_, …, *Y*_*n*_, *w*) and transmits (*σ*, *m*_*i*_, *t*_*i*_, *pk*_*i*_) to MS through public channels.
- **Verify-AS** (*params*, {*m*_*i*_, *t*_*i*_}_1 ≤ *i* ≤ *n*_, *σ*, *sk*_*ver*_)→ **VALID** or **INVALID**: Given *params*, *pk*_*i*_, {*m*_*i*_, *t*_*i*_}_1 ≤ *i* ≤ *n*_ and *σ*, MS performs the following procedures:
  - Computes *l*_*i*_ = *H*_1_(*R*_*i*_, *PID*_*i*_, *P*_*pub*_, *X*_*i*_) and *b*_*i*_ = *H*_2_(*PID*_*i*_, *m*_*i*_, *t*_*i*_, *pk*_*i*_, *Y*_*i*_).
  - Checks whether the equation $wP-\sum_{i=1}^{n}Y_{i}=\sum_{i=1}^{n}\left[b_{i}\left(X_{i}+R_{i}+l_{i}P_{pub}\right)\right]$ holds. If it holds, MS outputs **VALID** and accepts *σ*. Otherwise, MS outputs **INVALID** and rejects *σ*.

### Correctness

Given *params*, *pk*_*i*_, {*m*_*i*_, *t*_*i*_}_1 ≤ *i* ≤ *n*_ and *σ*_*i*_, the validity of the following equation is checked by CH.

$$\begin{array}{cc} W_{i}-Y_{i} & =\left[y_{i}+b_{i}\left(d_{i}+x_{i}\right)\right]P-Y_{i}\\ & =y_{i}P+b_{i}\left(d_{i}+x_{i}\right)P-Y_{i}\\ & =Y_{i}+b_{i}d_{i}P+b_{i}x_{i}P-Y_{i}\\ & =b_{i}\left(x_{i}+d_{i}\right)P\\ & =b_{i}\left(X_{i}+R_{i}+l_{i}P_{pub}\right). \end{array}$$

Given *params*, *pk*_*i*_, {*m*_*i*_, *t*_*i*_}_1 ≤ *i* ≤ *n*_ and *σ*, the validity of the following equation is checked by MS.

$$\begin{array}{cc} wP-\sum_{i=1}^{n}Y_{i} & =\sum_{i=1}^{n}\left[y_{i}+b_{i}\left(d_{i}+x_{i}\right)\right]P-\sum_{i=1}^{n}Y_{i}\\ & =\sum_{i=1}^{n}y_{i}P+\sum_{i=1}^{n}b_{i}\left(d_{i}P+x_{i}P\right)-\sum_{i=1}^{n}Y_{i}\\ & =\sum_{i=1}^{n}Y_{i}+\sum_{i=1}^{n}b_{i}\left(d_{i}P+x_{i}P\right)-\sum_{i=1}^{n}Y_{i}\\ & =\sum_{i=1}^{n}b_{i}\left(d_{i}P+x_{i}P\right)\\ & =\sum_{i=1}^{n}\left[b_{i}\left(X_{i}+R_{i}+l_{i}P_{pub}\right)\right]. \end{array}$$

### Security analysis

In this section, we give **Theorem 1** and **Theorem 2** to prove that our improved PF-CLAS scheme can resist malicious MSN_*i*_ attacks and malicious MS attacks.

**Theorem 1:** If $A_{1}$ (malicious MSN_*i*_) can successfully forge the signature in polynomial time with the non-negligible probability *ε*_1_, then there will be a challenger *ζ*_1_ that can work out the ECDLP with the probability $\left(1-\frac{1}{e}\right)\left(\frac{\varepsilon_{1}}{eq_{h_{i}}}\right)\left(1-\frac{1}{q_{ppk}+q_{v}+q_{s}+1}\right)$, where *e*, $q_{h_{i}}$, *q*_*s*_, *q*_*ppk*_, *q*_*v*_ are the natural logarithm base and the most times of *Hash Query*, *Signature Query*, *PPK Query*, *SV Query*.

**Proof:** The challenger *ζ*_1_ is a solver of the ECDLP. Given the tuple (*P*, *P*_*pub*_ = *sP*)∈*G*×*G*, the goal of *ζ*_1_ is to calculate $s\;\in \;Z_{q}^{*}$.

*Setup:* *ζ*_1_ performs **System Initialization** algorithm to generate *params* and *s*. *ζ*_1_ sends *params* to $A_{1}$ and keeps *s* secretly.

*Query Phase:* The challenger *ζ*_1_ cannot get the identity *PID*_*i*_ which is selected by $A_{1}$. Therefore, *ζ*_1_ guesses a random identity $PID_{i}^{*}$ as the identity, where *ζ*_1_ can correctly guess with probability $c=1-\frac{1}{q_{ppk}+q_{v}+q_{s}+1}$.

- *H*_1_ *Query:* *ζ*_1_ creates an empty *list*_1_. When receiving a query *H*_1_(*R*_*i*_, *PID*_*i*_, *P*_*pub*_, *X*_*i*_) from $A_{1}$, if there is a tuple (*R*_*i*_, *PID*_*i*_, *P*_*pub*_, *X*_*i*_, *l*_*i*_) in the *list*_1_, *ζ*_1_ will return *l*_*i*_ to $A_{1}$; Otherwise, *ζ*_1_ selects $l_{i}\;\in \;Z_{q}^{*}$ at random and adds the tuple (*R*_*i*_, *PID*_*i*_, *P*_*pub*_, *X*_*i*_, *l*_*i*_) into *list*_1_. Finally, *ζ*_1_ returns *l*_*i*_ to $A_{1}$.
- *H*_2_ *Query:* *ζ*_1_ creates an empty *list*_2_. When receiving a query *H*_2_(*m*_*i*_, *PID*_*i*_, *t*_*i*_, *pk*_*i*_, *Y*_*i*_) from $A_{1}$, if there is a tuple (*m*_*i*_, *PID*_*i*_, *t*_*i*_, *pk*_*i*_, *Y*_*i*_, *b*_*i*_) in the *list*_2_, *ζ*_1_ will return *b*_*i*_ to $A_{1}$; Otherwise, *ζ*_1_ selects $b_{i}\;\in \;Z_{q}^{*}$ at random and adds the tuple (*m*_*i*_, *PID*_*i*_, *t*_*i*_, *pk*_*i*_, *Y*_*i*_, *b*_*i*_) into *list*_2_. Finally, *ζ*_1_ returns *b*_*i*_ to $A_{1}$.
- *SV Query:* *ζ*_1_ creates an empty *list*_3_. When receiving a query about the secret value of MSN_*i*_ from $A_{1}$, if there is *x*_*i*_ in the *list*_3_, *ζ*_1_ will return *x*_*i*_ to $A_{1}$; Otherwise, *ζ*_1_ selects $x_{i}\;\in \;Z_{q}^{*}$ at random and adds *x*_*i*_ into *list*_3_. Finally, *ζ*_1_ returns *x*_*i*_ to $A_{1}$.
- *PPK Query:* *ζ*_1_ creates an empty *list*_4_. When receiving a query about the partial private key of MSN_*i*_ with *PID*_*i*_ from $A_{1}$, if there is a tuple (*R*_*i*_, *PID*_*i*_, *d*_*i*_) in the *list*_4_, *ζ*_1_ will return (*R*_*i*_, *d*_*i*_) to $A_{1}$; Otherwise, *ζ*_1_ queries the corresponding tuple (*R*_*i*_, *PID*_*i*_, *P*_*pub*_, *X*_*i*_, *l*_*i*_) of MSN_*i*_ with *PID*_*i*_ ∈ *list*_1_, selects $d_{i}\;\in \;Z_{q}^{*}$ at random, computes *R*_*i*_ = *d*_*i*_ *P* − *l*_*i*_ *P*_*pub*_ and adds the tuple (*R*_*i*_, *PID*_*i*_, *d*_*i*_) into *list*_4_. Finally, *ζ*_1_ returns (*R*_*i*_, *d*_*i*_) to $A_{1}$.
- *PK Query:* *ζ*_1_ creates an empty *list*_5_. When receiving a query about the public key of MSN_*i*_ with *PID*_*i*_ from $A_{1}$, if there is a tuple (*R*_*i*_, *PID*_*i*_, *X*_*i*_) in the *list*_5_, *ζ*_1_ will return (*R*_*i*_, *X*_*i*_) to $A_{1}$; Otherwise, *ζ*_1_ performs following steps.
  1. If $PID_{i}\neq PID_{i}^{*}$, *ζ*_1_ selects $x_{i},d_{i},l_{i}\;\in \;Z_{q}^{*}$ at random, computes *X*_*i*_ = *x*_*i*_ *P* and *R*_*i*_ = *d*_*i*_ *P* − *l*_*i*_ *P*_*pub*_. Then, *ζ*_1_ adds the tuple (*R*_*i*_, *PID*_*i*_, *X*_*i*_) into *list*_5_ and returns (*R*_*i*_, *X*_*i*_) to $A_{1}$.
  2. If $PID_{i}=PID_{i}^{*}$, *ζ*_1_ selects $x_{i},r_{i}\;\in \;Z_{q}^{*}$ at random, computes *X*_*i*_ = *x*_*i*_ *P* and *R*_*i*_ = *r*_*i*_ *P*. Then, *ζ*_1_ sets *d*_*i*_ as ⊥ and adds the tuple (*R*_*i*_, *PID*_*i*_, *X*_*i*_) into *list*_5_. Finally, it returns (*R*_*i*_, *X*_*i*_) to $A_{1}$.
- *PK Replacement Query:* When $A_{1}$ selects a new public key $pk_{i}^{*}=\left(X_{i}^{*},R_{i}^{*}\right)$ and sends $\left(PID_{i},pk_{i}^{*}\right)$ to *ζ*_1_. When receiving a query about the public key replacement of MSN_*i*_ with *PID*_*i*_ from $A_{1}$, *ζ*_1_ updates *list*_5_ and records this replacement.
- *Signature Query:* *ζ*_1_ creates an empty *list*_6_. When receiving a query about the signature of MSN_*i*_ with *PID*_*i*_ from $A_{1}$, if there is a tuple (*m*_*i*_, *PID*_*i*_, *x*_*i*_, *ω*_*i*_) in the *list*_6_, *ζ*_1_ selects $y_{i}\;\in \;Z_{q}^{*}$ at random, computes *Y*_*i*_ = *y*_*i*_ *P*, *b*_*i*_ = *H*_2_(*PID*_*i*_, *m*_*i*_, *t*_*i*_, *Y*_*i*_, *pk*_*i*_) and *w*_*i*_ = *y*_*i*_ + *b*_*i*_(*x*_*i*_ + *d*_*i*_) mod *q*. Then *ζ*_1_ returns (*Y*_*i*_, *w*_*i*_) to $A_{1}$; Otherwise, *ζ*_1_ selects $w_{i}\;\in \;Z_{q}^{*}$ at random, computes *Y*_*i*_ = *w*_*i*_ *P*−*b*_*i*_(*X*_*i*_ + *R*_*i*_ + *l*_*i*_ *P*_*pub*_) and adds the tuple (*Y*_*i*_, *w*_*i*_) into *list*_6_. Finally, *ζ*_1_ returns (*Y*_*i*_, *w*_*i*_) to $A_{1}$.

*Forgery:* After polynomial bounded times of queries, $A_{1}$ outputs forged signature $\sigma_{i}^{*}=\left(Y_{i}^{*},w_{i}^{*}\right)$ under the tuple $\left(PID_{i}^{*},m_{i}^{*},t_{i}^{*},X_{i}^{*}\right)$. According to the forking lemma [32], $A_{1}$ generates another forged signature $\sigma_{i}^{*(2)}=\left(Y_{i}^{*(2)},w_{i}^{*(2)}\right)$. Therefore, according to the equation $w_{i}^{*}P=Y_{i}^{*}+b_{i}^{*}\left(X_{i}^{*}+R_{i}^{*}+l_{i}^{*}P_{pub}\right)$ and the equation $w_{i}^{*(2)}P=Y_{i}^{*(2)}+b_{i}^{*(2)}\left(X_{i}^{*(2)}+R_{i}^{*(2)}+l_{i}^{*}P_{pub}\right)$, *s* can be obtained as a valid solution. Otherwise, *ζ*_1_ cannot handle the ECDLP.

In order to succeed in forging a signature, the outputs of *ζ*_1_ need to satisfy the following conditions:

1. *T*_1_: *ζ*_1_ has never aborted the process of quering;
2. *T*_2_: *ζ*_1_ has never aborted the process of forging the signature;
3. *T*_3_: $\sigma_{i}^{*}$ is a valid signature.

According to the above conditions, we can get that *P*_*r*_[*T*_1_] ≥ 1 − *c*, $P_{r}\left[T_{1}|T_{2}\right]\geq \left(1-c\right)c^{q_{ppk}+q_{v}+q_{s}}$ and $P_{r}\left[T_{1}|T_{2}\wedge T_{3}\right]\geq \left(1-c\right)c^{q_{ppk}+q_{v}+q_{s}}\left(1-\frac{1}{e}\right)\frac{\varepsilon_{1}}{q_{h_{i}}}\geq \left(1-\frac{1}{e}\right)\left(\frac{\varepsilon_{1}}{eq_{h_{i}}}\right)\left(1-\frac{1}{q_{ppk}+q_{v}+q_{s}+1}\right)$. Consequently, the probability that *ζ*_1_ can work out the ECDLP is $\left(1-\frac{1}{e}\right)\left(\frac{\varepsilon_{1}}{eq_{h_{i}}}\right)\left(1-\frac{1}{q_{ppk}+q_{v}+q_{s}+1}\right)$.

**Theorem 2:** If $A_{2}$ (malicious MS) can successfully forge the signature in polynomial time with the non-negligible probability *ε*_2_, then there will be a challenger *ζ*_2_ that can work out the ECDLP with the probability $\left(1-\frac{1}{e}\right)\left(\frac{\varepsilon_{2}}{eq_{h_{i}}}\right)\left(1-\frac{1}{q_{v}+q_{s}+1}\right)$, where *e*, $q_{h_{i}}$, *q*_*s*_, *q*_*v*_ are the natural logarithm base and the most times of *Hash Query*, *Signature Query*, *SV Query*.

**Proof:** The challenger *ζ*_2_ is a solver of the ECDLP. Given the tuple (*P*, *X*_*i*_ = *x*_*i*_ *P*) ∈ *G* × *G*, the goal of *ζ*_2_ is to calculate $x_{i}\;\in \;Z_{q}^{*}$.

*Setup:* *ζ*_2_ performs **System Initialization** algorithm to generate *params* and *s*. *ζ*_2_ sends *params* and *s* to $A_{2}$.

*Query Phase:* The challenger *ζ*_2_ cannot get the identity *PID*_*i*_ which is selected by $A_{2}$. Therefore, *ζ*_2_ guesses a random identity $PID_{i}^{*}$ as the identity, where *ζ*_2_ can correctly guess with probability $c=1-\frac{1}{q_{v}+q_{s}+1}$.

- *H*_1_ *Query:* *ζ*_2_ creates an empty *list*_1_. When receiving a query *H*_1_(*R*_*i*_, *PID*_*i*_, *P*_*pub*_, *X*_*i*_) from $A_{2}$, if there is a tuple (*R*_*i*_, *PID*_*i*_, *P*_*pub*_, *X*_*i*_, *l*_*i*_) in the *list*_1_, *ζ*_2_ will return *l*_*i*_ to $A_{2}$; Otherwise, *ζ*_2_ selects $l_{i}\;\in \;Z_{q}^{*}$ at random and adds the tuple (*R*_*i*_, *PID*_*i*_, *P*_*pub*_, *X*_*i*_, *l*_*i*_) into *list*_1_. Finally, *ζ*_2_ returns *l*_*i*_ to $A_{2}$.
- *H*_2_ *Query:* *ζ*_2_ creates an empty *list*_2_. When receiving a query *H*_2_(*m*_*i*_, *PID*_*i*_, *t*_*i*_, *pk*_*i*_, *Y*_*i*_) from $A_{2}$, if there is a tuple (*m*_*i*_, *PID*_*i*_, *t*_*i*_, *pk*_*i*_, *Y*_*i*_, *b*_*i*_) in the *list*_2_, *ζ*_2_ will return *b*_*i*_ to $A_{2}$; Otherwise, *ζ*_2_ selects $b_{i}\;\in \;Z_{q}^{*}$ at random and adds the tuple (*m*_*i*_, *PID*_*i*_, *t*_*i*_, *pk*_*i*_, *Y*_*i*_, *b*_*i*_) into *list*_2_. Finally, *ζ*_2_ returns *b*_*i*_ to $A_{2}$.
- *SV Query:* *ζ*_2_ creates an empty *list*_3_. When receiving a query about the secret value of MSN_*i*_ from $A_{2}$, if there is *x*_*i*_ in the *list*_3_, *ζ*_2_ will return *x*_*i*_ to $A_{2}$; Otherwise, *ζ*_2_ selects $x_{i}\;\in \;Z_{q}^{*}$ at random and adds the tuple *x*_*i*_ into *list*_3_. Finally, *ζ*_2_ returns *x*_*i*_ to $A_{2}$.
- *PK Query:* *ζ*_2_ creates an empty *list*_4_. When receiving a query about the public key of MSN_*i*_ with *PID*_*i*_ from $A_{2}$, if there is a tuple (*R*_*i*_, *PID*_*i*_, *X*_*i*_) in the *list*_4_, *ζ*_2_ will return (*R*_*i*_, *X*_*i*_) to $A_{2}$; Otherwise, *ζ*_2_ performs following steps.
  1. If $PID_{i}\neq PID_{i}^{*}$, *ζ*_2_ selects $x_{i},d_{i},l_{i}\;\in \;Z_{q}^{*}$ at random, computes *X*_*i*_ = *x*_*i*_ *P* and *R*_*i*_ = *d*_*i*_ *P*−*l*_*i*_ *P*_*pub*_. Then, *ζ*_2_ adds the tuple (*R*_*i*_, *PID*_*i*_, *X*_*i*_) into *list*_4_ and returns (*R*_*i*_, *X*_*i*_) to $A_{2}$.
  2. If $PID_{i}=PID_{i}^{*}$, *ζ*_2_ selects $x_{i},r_{i}\;\in \;Z_{q}^{*}$ at random, computes *X*_*i*_ = *x*_*i*_ *P* and *R*_*i*_ = *r*_*i*_ *P*. Then, *ζ*_2_ sets *d*_*i*_ as ⊥ and adds the tuple (*R*_*i*_, *PID*_*i*_, *X*_*i*_) into *list*_4_. Finally, it returns (*R*_*i*_, *X*_*i*_) to $A_{2}$.
- *Signature Query:* *ζ*_2_ creates an empty *list*_5_. When receiving a query about the signature of MSN_*i*_ with *PID*_*i*_ from $A_{2}$, if there is a tuple (*m*_*i*_, *PID*_*i*_, *x*_*i*_, *ω*_*i*_) in the *list*_5_, *ζ*_2_ selects $y_{i}\;\in \;Z_{q}^{*}$ at random, computes *Y*_*i*_ = *y*_*i*_ *P*, *b*_*i*_ = *H*_2_(*PID*_*i*_, *m*_*i*_, *t*_*i*_, *Y*_*i*_, *pk*_*i*_) and *w*_*i*_ = *y*_*i*_ + *b*_*i*_(*x*_*i*_ + *d*_*i*_) mod *q*. Then *ζ*_2_ returns (*Y*_*i*_, *w*_*i*_) to $A_{2}$; Otherwise, *ζ*_2_ selects $w_{i}\;\in \;Z_{q}^{*}$ at random, computes *Y*_*i*_ = *w*_*i*_ *P*−*b*_*i*_(*X*_*i*_ + *R*_*i*_ + *l*_*i*_ *P*_*pub*_) and adds the tuple (*Y*_*i*_, *w*_*i*_) into *list*_5_. Finally, *ζ*_2_ returns (*Y*_*i*_, *w*_*i*_) to $A_{2}$.

*Forgery:* After polynomial bounded times of queries, $A_{2}$ outputs forged signature $\sigma_{i}^{*}=\left(Y_{i}^{*},w_{i}^{*}\right)$ under the tuple $\left(PID_{i}^{*},m_{i}^{*},t_{i}^{*},R_{i}^{*}\right)$. According to the forking lemma [32], $A_{2}$ generates another forged signature $\sigma_{i}^{*(2)}=\left(Y_{i}^{*(2)},w_{i}^{*(2)}\right)$. Therefore, according to the equation $w_{i}^{*}P=Y_{i}^{*}+b_{i}^{*}\left(X_{i}+R_{i}^{*}+h_{i}^{*}P_{pub}\right)$ and the equation $w_{i}^{*(2)}P=Y_{i}^{*(2)}+b_{i}^{*(2)}\left(X_{i}+R_{i}^{*(2)}+h_{i}^{*(2)}P_{pub}\right)$, *x*_*i*_ can be obtained as a valid solution. Otherwise, *ζ*_2_ cannot handle the ECDLP.

In order to succeed in forging a signature, the outputs of *ζ*_2_ need to satisfy the following conditions:

1. *T*_1_: *ζ*_2_ has never aborted the process of quering;
2. *T*_2_: *ζ*_2_ has never aborted the process of forging the signature;
3. *T*_3_: $\sigma_{i}^{*}$ is a valid signature.

According to the above conditions, we can get that *P*_*r*_[*T*_1_]≥1−*c*, $P_{r}\left[T_{1}|T_{2}\right]\geq \left(1-c\right)c^{q_{v}+q_{s}}$ and $P_{r}\left[T_{1}|T_{2}\wedge T_{3}\right]\geq \left(1-c\right)c^{q_{v}+q_{s}}\left(1-\frac{1}{e}\right)\frac{\varepsilon_{2}}{q_{h_{i}}}\geq \left(1-\frac{1}{e}\right)\left(\frac{\varepsilon_{2}}{eq_{h_{i}}}\right)\left(1-\frac{1}{q_{v}+q_{s}+1}\right)$. Consequently, the probability that *ζ*_2_ can work out the ECDLP is $\left(1-\frac{1}{e}\right)\left(\frac{\varepsilon_{2}}{eq_{h_{i}}}\right)\left(1-\frac{1}{q_{v}+q_{s}+1}\right)$.

### Other security analysis

1. *Message authentication and integrity:* According to **Theorem 1** and **Theorem 2**, neither Type I nor Type II attackers can pass the verification by forging a signature.
2. *Anonymity:* In the improved PF-CLAS scheme, *PID*_*i*_ is the pseudo identity of MSN_*i*_, where *PID*_*i*_ = *RID*_*i*_⊕*H*(*r*_*i*_ *P*_*pub*_, *T*_*i*_). Any adversary cannot extract the real identity of MSN_*i*_. Hence, our scheme provides strong anonymity.
3. *Traceability:* If MSN_*i*_ transmits illegal information, MS can track abnormal MSN_*i*_ and extract its real identity by computing *RID*_*i*_ = *PID*_*i*_⊕*H*(*sR*_*i*_), where *sR*_*i*_ = *r*_*i*_ *P*_*pub*_.

## Performance evaluation

In this section, we will provide the performance analysis in terms of computational overhead, communication overhead, and security features. In the meantime, the efficiency of the improved scheme will be compared with the related schemes [15, 24, 25, 27, 29, 33, 34]. We utilize MIRACL library to simulate cryptographic operations on a Windows 10 laptop with an Intel i7–1195G7 @2.9 GHz processor and 8 GB of memory. The measured runtime of different operations is shown in Table 2.

**Table 2:** Runtime of cryptographic operations.

Operations	Abbreviations	Runtime (ms)
Pairing-based scalar multiplication	*T*_*sm*_	2.2560
Pairing-based point addition	*T*_*pa*_	0.1732
Bilinear pairing computation	*T*_*p*_	4.6028
Map-to-point hash	*T*_*h*_	5.1240
ECC-based scalar multiplication	*T*_*esm*_	0.7648
ECC-based point addition	*T*_*epa*_	0.0435

### Computational overhead

**Fig 2:**
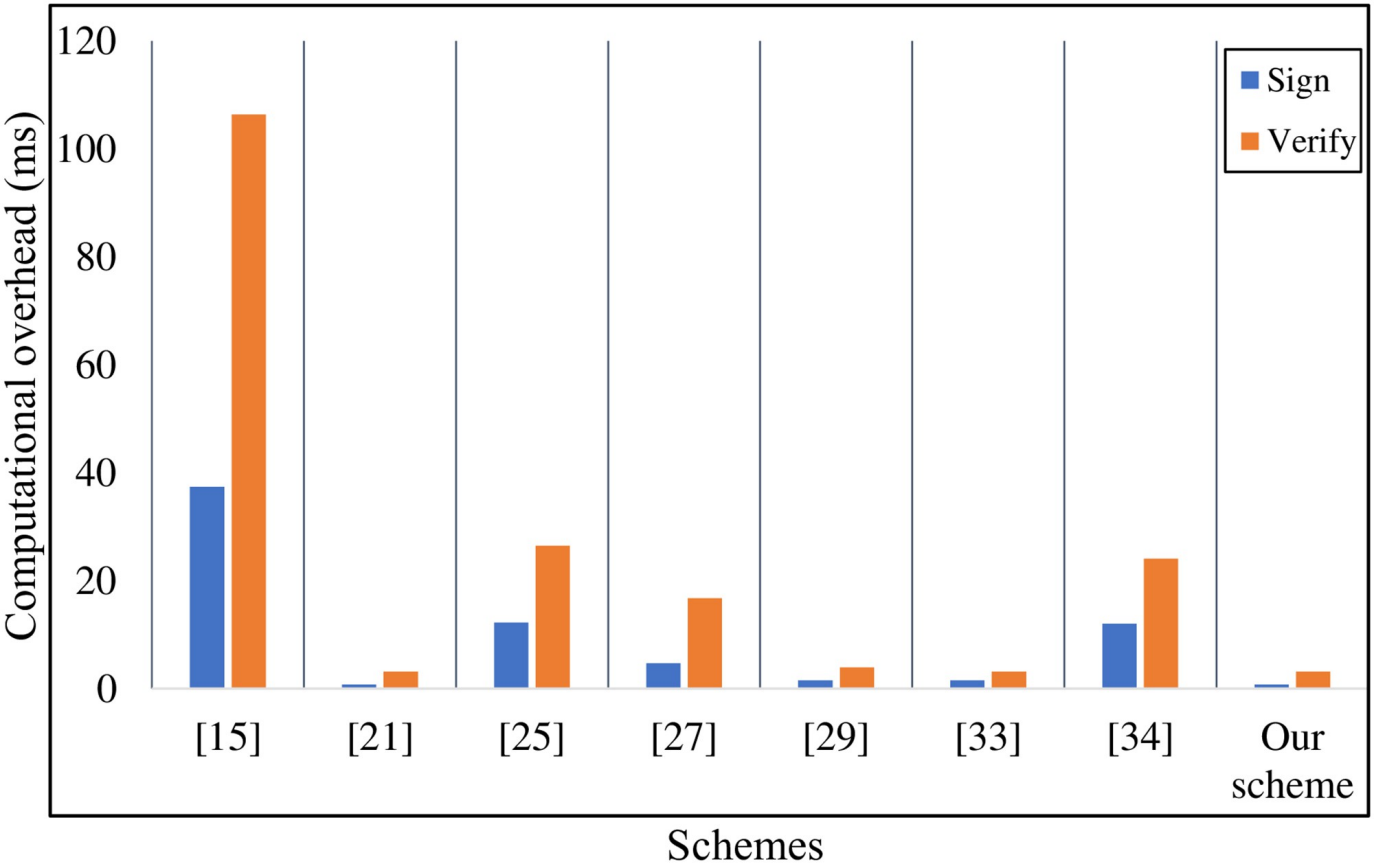
Computational overhead of the single signing and verification.

**Fig 3:**
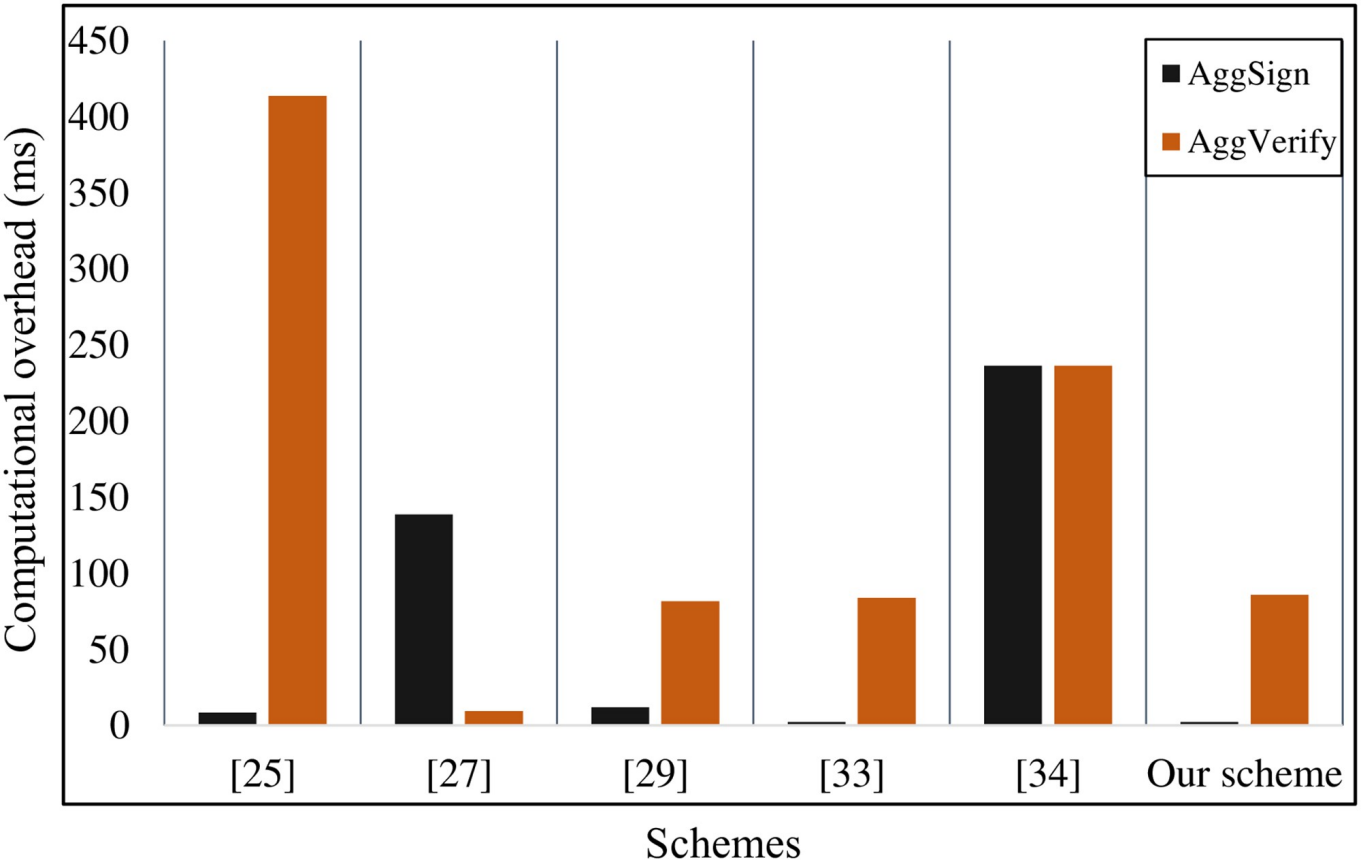
Computational overhead of the aggregate signing and aggregate verification.

As is described in Table 3, we mainly count the computational overhead of **Generate-Signature** algorithm, **Verify-Signature** algorithm, **Generate-AS** algorithm, and **Verify-AS** algorithm. In Xu *et al*.’s scheme [15], the computational overhead of the single signing and verification is ≈ 143.7864 ms. Similarly, Kumar *et al*.’s scheme [25], Liu *et al*.’s scheme [27], and Shen *et al*.’s scheme [34] need 38.724 ms, 21.444ms, 36.1212 ms, respectively. As is shown in Fig 2 the computational overhead of the above schemes is extremely high. The root cause is that these schemes all use bilinear pairing and map-to-point hash operations to construct the signature. Hence, we use pairing-free operations to improve the efficiency of the improved PF-CLAS scheme. In literatures [24, 29, 33], their schemes also don’t use bilinear pairings. Hence, they only need 3.9545 ms, 5.4841 ms, 4.1793 ms, respectively. The computational overhead of the single signing and verification only needs 3.9545ms, which saves 97.2%, 89.8%, 81.6%, 27.9%, 5.4%, 89.1% of the computational overhead than Xu *et al*.’s scheme [15], Kumar *et al*.’s scheme [25], Liu *et al*.’s scheme [27], Gayathri *et al*.’s scheme [29], Verma *et al*.’s scheme [33], Shen *et al*.’s scheme [34]. In the aggregate signing and aggregate verification phases, we set the number of signatures participating in the aggregation as *n* = 50. Since references [15, 24] have no connection with the aggregate signature, we don’t describe them too much. As is shown in Fig 3, the computational overhead of the aggregate signing and verification of Kumar *et al*.’s scheme [25], Liu *et al*.’s scheme [27], Gayathri *et al*.’s scheme [29], Verma *et al*.’s scheme [33], Shen *et al*.’s scheme [34] is 422.0506 ms, 147.9858 ms, 95.5493 ms, 85.9013 ms, 472.56 ms, respectively. Our improved PF-CLAS scheme needs 88.0328 ms, which saves 79.2%, 41%, 7.9%, 27.9%, 5.4%, 81.4% than Kumar *et al*.’s scheme [25], Liu *et al*.’s scheme [27], Gayathri *et al*.’s scheme [29], Shen *et al*.’s scheme [34]. Although the total computational overhead of Verma *et al*.’s scheme [33] is basically the same as our scheme, Verma *et al*.’s scheme [33] cannot achieve secure communication. Hence, the computational overhead of our improved PF-CLAS scheme reaches the upstream level of the relevant schemes.

**Table 3:** Comparison of computational overhead.

Schemes	Sign (ms)	Verify (ms)	AggregateSign (ms)	AggregateVerify (ms)
[15]	14*T*_*sm*_+4*T*_*pa*_+*T*_*h*_ ≈ 37.4008	22*T*_*p*_+*T*_*h*_ ≈ 106.3856	—	—
[24]	*T*_*esm*_ ≈ 0.7648	4*T*_*esm*_+3*T*_*epa*_ ≈ 3.1897	—	—
[25]	3*T*_*sm*_+2*T*_*pa*_+*T*_*h*_ ≈ 12.2384	3*T*_*p*_+*T*_*sm*_+*T*_*pa*_+2*T*_*h*_ ≈ 26.4856	(*n*−1)*T*_*pa*_ ≈ 8.4846	3*T*_*p*_+*nT*_*sm*_+(3*n*−2)*T*_*pa*_+(*n*+ 1)*T*_*h*_ ≈ 413.566
[27]	2*T*_*sm*_+*T*_*pa*_ ≈ 4.6852	2*T*_*p*_+*T*_*sm*_+*T*_*pa*_+*T*_*h*_ ≈ 16.7588	*nT*_*sm*_+(3*n*−1)*T*_*pa*_ ≈ 138.607	2*T*_*p*_+*T*_*pa*_ ≈ 9.3788
[29]	2*T*_*esm*_ ≈ 1.5296	5*T*_*esm*_+3*T*_*epa*_ ≈ 3.9545	2*nT*_*esm*_+(2*n*−2)*T*_*epa*_ ≈ 11.911	(2*n*+ 1)*T*_*esm*_+(2*n*+ 1)*T*_*epa*_ ≈ 81.6383
[33]	2*T*_*esm*_ ≈ 1.5296	4*T*_*esm*_+3*T*_*epa*_ ≈ 3.1897	(*n*−1)*T*_*epa*_ ≈ 2.1315	(2*n*+ 1)*T*_*esm*_+3*nT*_*epa*_ ≈ 83.7698
[34]	3*T*_*sm*_+*T*_*pa*_+ *T*_*h*_ ≈ 12.0652	3*T*_*p*_+2*T*_*h*_ ≈ 24.0560	*nT*_*p*_+*T*_*sm*_+ (*n*−1)*T*_*pa*_ ≈ 236.28	*nT*_*p*_+*T*_*sm*_+ (*n*−1)*T*_*pa*_ ≈ 236.28
Our scheme	*T*_*esm*_ ≈ 0.7648	4*T*_*esm*_+3*T*_*epa*_ ≈ 3.1897	(*n*−1)*T*_*epa*_ ≈ 2.1315	(2*n*+ 1)*T*_*esm*_+(4*n*−1)*T*_*epa*_ ≈ 85.9013

We set the number of signatures participating in the aggregation as *n* = 50.

### Communication overhead

**Fig 4:**
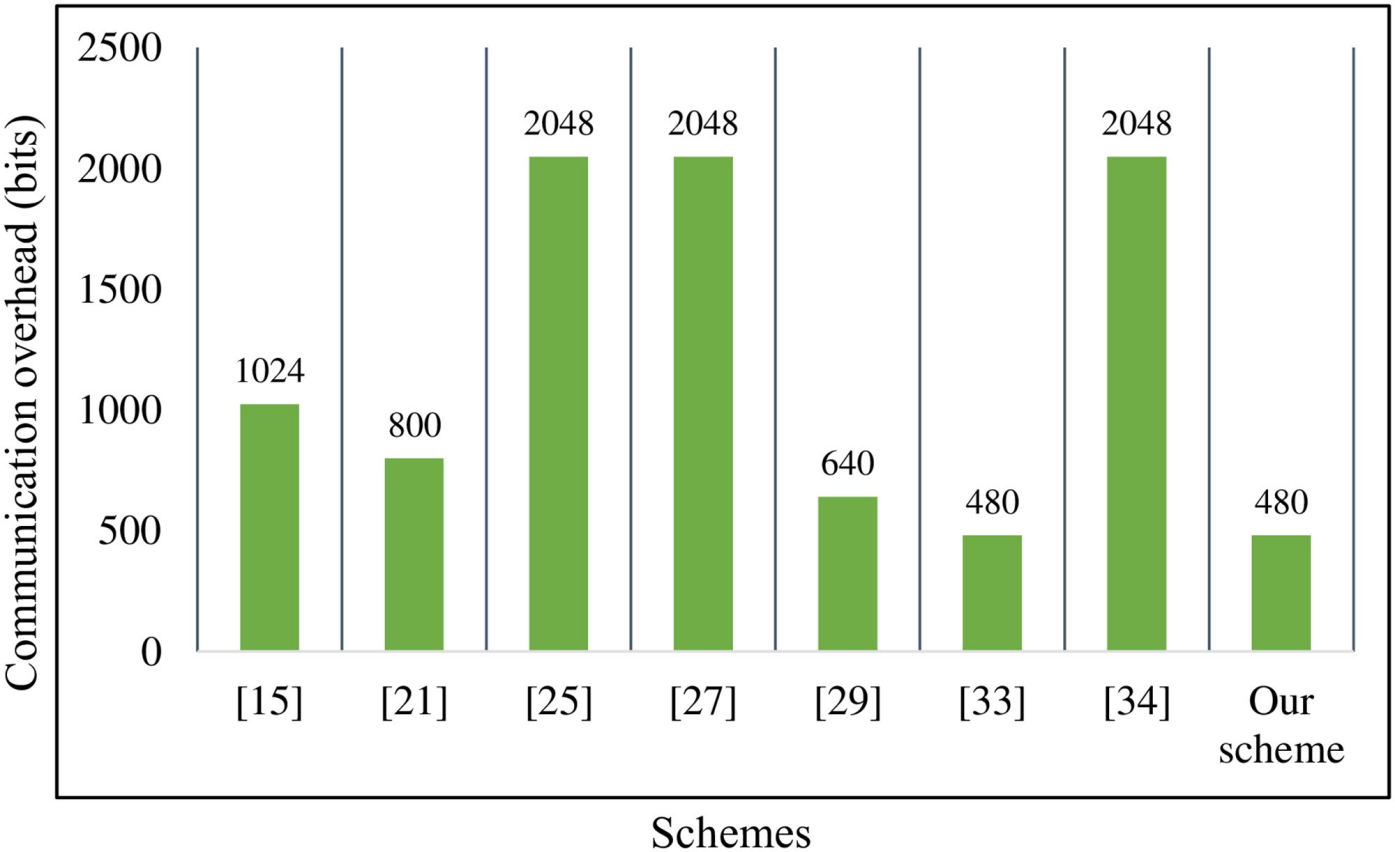
Communication overhead of single signatures.

**Fig 5:**
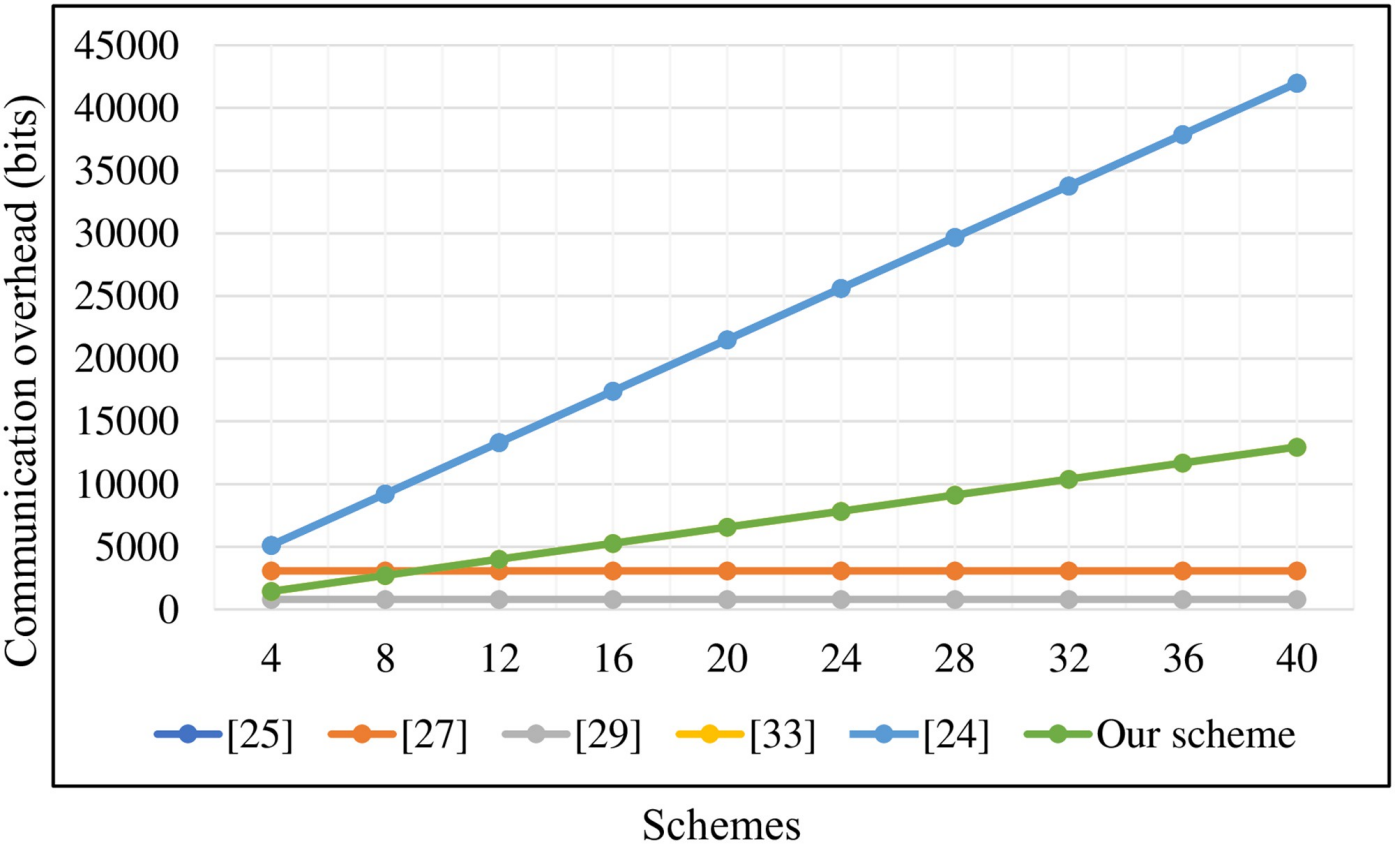
Communication overhead of aggregate signatures.

As shown in Table 4, we list parameters and length specifications for pairing-based and ECC-based schemes [29]. In addition, the size of the group $|Z_{q}^{*}|$ is 160 bits in our scheme. In [15, 25, 27, 34], the communication overhead of the single signature is 1024 bits, 2048 bits, 2048 bits, and 2048 bits, respectively, because all the elements of *σ*_*i*_ belong to *G*_1_. In our improved PF-CLAS, we set *σ*_*i*_ as (*Y*_*i*_, *w*_*i*_), where *Y*_*i*_ ∈ *G*, $w_{i}\;\in \;Z_{q}^{*}$. Compared with the schemes [15, 24, 25, 27, 29, 34], the communication overhead of the single signature in our scheme is reduced by 53.1%, 40%, 76.57%, 76.57%, 25%, 76.57%. As is described in Fig 4, it is obvious that our scheme has higher efficiency than the above schemes in the single signature phase. Since references [15, 24] have no connection with the aggregate signature, we don’t describe them too much in the aggregate signature phase. In the meantime, we can know from Fig 5 that the communication overhead of the aggregate signatures in our scheme is lower than Kumar *et al*.’s scheme [25] and Shen *et al*.’s scheme [34] with the increase of the number of medical sensor nodes. Although Liu *et al*.’s scheme [27] and Gayathri *et al*.’s scheme [29] have lower communication overhead than our scheme, their schemes have serious security flaws. As shown in Table 5, even though our scheme has the same communication overhead as Verma *et al*.’s scheme [33], their scheme cannot meet the security requirements of HWMSNs. Therefore, our scheme has certain advantages in terms of communication overhead.

**Table 4:** Length of parameters in bilinear pairing and ECC.

Type of the scheme	Type of the curve	Pairing	Cyclic group	Size of the prime	Size of the group
Bilinear Pairing	*E*: *y*^2^ = *x*^3^+ *x* mod *p*	*e*: *G*_1_×*G*_1_ → *G*_*T*_	*G*_1_(*P*)	*p*=512 bits	|*G*_1_|=1024 bits
ECC	*E*: *y*^2^ = *x*^3^+ *ax*+ *b* mod *p*	—	*G*(*P*)	*p*=160 bits	|*G*|=320 bits

**Table 5:** Comparison of communication overhead and security features.

Schemes	Single signatures	Aggregate signatures	Type I attacks	Type II attacks	Anonymity	Traceability
[15]	|*G*_1_| = 1024 bits	—	—	—	×	×
[24]	$\left|G|+3\right|Z_{q}^{*}|=800$ bits	—	✔	✔	×	×
[25]	2|*G*_1_| = 2048 bits	(*n*+1)|*G*_1_| = 1024(*n*+1) bits	✔	×	×	×
[27]	2|*G*_1_| = 2048 bits	3|*G*_1_| = 3072 bits	×	×	✔	×
[29]	$\left|G|+2\right|Z_{q}^{*}|=640$ bits	$2|G|+|Z_{q}^{*}|=800$ bits	×	×	✔	✔
[33]	$\left|G|+\right|Z_{q}^{*}|=480$ bits	$n|G|+|Z_{q}^{*}|=160(2n+1)$ bits	×	✔	×	×
[34]	2|*G*_1_| = 2048 bits	(*n*+1)|*G*_1_| = 1024(*n*+1) bits	✔	✔	×	×
Our scheme	$\left|G|+\right|Z_{q}^{*}|=480$ bits	$n|G|+|Z_{q}^{*}|=160(2n+1)$ bits	✔	✔	✔	✔

### Security features

As shown in Table 5, Xu *et al*.’s scheme [15], Xu *et al*.’s scheme [24], Kumar *et al*.’s scheme [25], Verma *et al*.’s scheme [33], and Shen *et al*.’s scheme [34] don’t consider the anonymity of patients’ identities and tracing of malicious medical sensor nodes, which are unsuitable for HWMSNs scenarios. Although Liu *et al*.’s scheme [27] and Gayathri *et al*.’s scheme [29] can meet the security requirements of HWMSNs, these schemes have security drawbacks that cannot withstand Type I and Type II attacks. The proposed scheme has been proved that resist Type I and Type II attacks under the random oracle model. Besides, our scheme is able to realize anonymity and traceability, which is more practical in HWMSNs.

## Conclusion

In this paper, we found that Zhan *et al*.’s PF-CLAS scheme [31] cannot withstand malicious MSN_*i*_ attacks. In the meantime, we showed the reason why this scheme was vulnerable to malicious MSN_*i*_ attacks. It is obvious that Zhan *et al*.’s scheme cannot guarantee the identity privacy of patients and secure transmission of medical data. Hence, we gave methods to fix the vulnerability and constructed an improved PF-CLAS scheme that could ensure provable security. In addition, the performance evaluation indicated that our improved scheme can realize privacy preservation and secure communication at low overhead. In the future, how to combine blockchain and edge computing technologies to design a more lightweight and secure CLAS scheme for HWMSNs is still an interesting problem.

## Supporting information

S1 Data Runtime of cryptographic operations. (XLS)

S2 Data Comparison of computational overhead. (XLS)
